# Decreased Serum Salusin-β Levels Are Independently Associated with Gestational Diabetes Mellitus

**DOI:** 10.3390/biomedicines14071602

**Published:** 2026-07-17

**Authors:** Hanise Ozkan Sonay, Yildiz Okuturlar, Esra Cokicli, Berrak Sahtiyanci, Irem Kirac Utku, Kerem Doga Seckin, Iskender Ekinci, Eda Nur Duran, Gulden Anataca, Burak Onal, Abdulbaki Kumbasar, Murat Akarsu, Omur Tabak

**Affiliations:** 1 Department of Internal Medicine, Kanuni Sultan Suleyman Training and Research Hospital, 34303 Istanbul, Türkiye; haniseozkan@hotmail.com (H.O.S.); muratakarsu79@gmail.com (M.A.);; 2 Department of Internal Medicine, Acibadem Mehmet Ali Aydinlar University School of Medicine, 34752 Istanbul, Türkiye; 3 Department of Internal Medicine, Faculty of Medicine, Istanbul Medipol University, 34214 Istanbul, Türkiye; 4 Department of Internal Medicine, Istanbul Physical Therapy and Rehabilitation Training and Research Hospital, 34182 Istanbul, Türkiye; 5 Department of Geriatrics, Tekirdag Dr. Ismail Fehmi Cumalioglu City Hospital, 59030 Tekirdag, Türkiye; 6 Department of Obstetrics and Gynecology, Faculty of Medicine, Istinye University, 34396 Istanbul, Türkiye; 7 Department of Internal Medicine, Faculty of Medicine, Bezmialem Vakıf University Hospital, 34093 Istanbul, Türkiye; 8 Department of Internal Medicine, Pervari State Hospital, 56700 Siirt, Türkiye; enurduran@gmail.com; 9 Department of Nursing Services, Kanuni Sultan Suleyman Training and Research Hospital, 34303 Istanbul, Türkiye; 10 Department of Medical Pharmacology, Faculty of Medicine, Biruni University, 34015 Istanbul, Türkiye; 11 Department of Internal Medicine, Bakirkoy Dr. Sadi Konuk Training and Research Hospital, 34147 Istanbul, Türkiye

**Keywords:** gestational diabetes mellitus, biomarkers, insulin resistance, endothelial dysfunction, inflammation, pregnancy, glucose intolerance, oxidative stress, cardiometabolic risk, salusin-β

## Abstract

**Background:** Gestational diabetes mellitus (GDM) is characterized by pregnancy-induced insulin resistance and β-cell dysfunction and is increasingly recognized as a state of cardiometabolic and endothelial dysregulation. Salusin-β, a bioactive peptide implicated in vascular inflammation and metabolic disorders, may play a role in GDM pathophysiology. However, data regarding its clinical relevance in GDM remain limited. **Objective**: This study aimed to compare serum salusin-β levels between women with GDM and healthy pregnant controls and to evaluate its diagnostic performance. **Methods:** This prospective study included 144 pregnant women between 24 and 28 weeks of gestation (70 with GDM and 74 healthy controls). GDM was diagnosed using a 75-g oral glucose tolerance test according to American Diabetes Association criteria. Serum salusin-β levels were measured using ELISA. Between-group comparisons were performed using appropriate parametric or non-parametric tests. Multivariable logistic regression analysis was performed to evaluate the independent association between salusin-β and GDM. Sensitivity analyses using an expanded adjustment model were also conducted. Diagnostic performance was evaluated using receiver operating characteristic (ROC) curve analysis. **Results:** Salusin-β levels were significantly lower in women with GDM compared to controls (68.53 [53.05–84.27] vs. 128.03 [76.74–261.35] pg/mL; *p* < 0.001). In the expanded multivariable logistic regression analysis, lower serum salusin-β levels remained independently associated with GDM (OR = 0.990, 95% CI: 0.985–0.996, *p* = 0.001). ROC analysis demonstrated acceptable discriminatory performance (AUC = 0.754, 95% CI: 0.672–0.833). The optimal cut-off value of 87.1 pg/mL yielded 80.0% sensitivity and 66.2% specificity. **Conclusions:** Serum salusin-β levels are significantly reduced in women with GDM and independently associated with disease presence. Although not sufficient as a standalone diagnostic marker, salusin-β demonstrates moderate discriminatory ability and may serve as a complementary biomarker reflecting vascular and metabolic dysregulation in GDM.

## 1. Introduction

Gestational diabetes mellitus (GDM) is one of the most common metabolic complications of pregnancy, defined as glucose intolerance with onset or first recognition during gestation. Its prevalence varies according to diagnostic criteria and population characteristics, ranging from approximately 7% to 18% worldwide, with an increasing trend paralleling the global rise in obesity and sedentary lifestyles [1,2,3]. Beyond adverse obstetric outcomes such as macrosomia and preeclampsia, GDM is associated with long-term metabolic and cardiovascular risks for both the mother and offspring, including an increased risk of type 2 diabetes mellitus and cardiovascular disease [3]. Early identification of affected pregnancies is therefore essential to mitigate both short- and long-term complications.

The pathophysiology of GDM is characterized by pancreatic β-cell dysfunction superimposed on progressively increasing pregnancy-induced insulin resistance [4]. Beyond these classical metabolic alterations, accumulating evidence indicates that GDM involves a complex interplay of metabolic, inflammatory, and vascular mechanisms. Chronic low-grade inflammation, dysregulated adipokine secretion, and enhanced oxidative stress have been shown to contribute to endothelial activation and vascular dysfunction in pregnancies complicated by GDM [5,6]. These alterations are accompanied by increased circulating pro-inflammatory mediators and impaired endothelial nitric oxide bioavailability, which collectively promote endothelial dysfunction and vascular imbalance during gestation [7,8].

Moreover, placental dysfunction and altered angiogenic signaling have been implicated in the pathogenesis of GDM, linking maternal metabolic stress with impaired placental perfusion and endothelial homeostasis [9]. Oxidative stress and endothelial injury may further exacerbate insulin resistance and disrupt maternal–fetal metabolic adaptation, thereby contributing to both short-term obstetric complications and long-term cardiometabolic risk [10,11]. Taken together, these findings suggest that GDM should not be regarded solely as a disorder of glucose metabolism but rather as a state of cardiometabolic and endothelial dysregulation characterized by intertwined inflammatory, vascular, and metabolic pathways.

In line with this evolving pathophysiological perspective, increasing attention has been directed toward circulating biomarkers reflecting endothelial dysfunction, inflammatory activation, and cardiometabolic stress, which may provide additional prognostic and mechanistic value beyond traditional glycemic indices [12].

Given that GDM is increasingly recognized as a state of endothelial dysfunction and cardiometabolic stress characterized by inflammatory activation and vascular imbalance, it is biologically plausible that vascular-related peptides may be altered in pregnancies complicated by GDM. Considering the established role of salusin-β in vascular inflammation and atherogenic processes, circulating salusin-β levels may be affected in the setting of GDM-related metabolic and endothelial dysregulation.

Salusin-β is a 20-amino-acid bioactive peptide derived from the prosalusin precursor and widely expressed in the central nervous system, vascular endothelium, and endocrine tissues [13]. Experimental studies have demonstrated that salusin-β exerts vasoactive and proatherogenic effects through modulation of inflammatory signaling pathways, endothelial function, and macrophage foam cell formation [14]. Moreover, altered circulating salusin-β levels have been reported in various metabolic and cardiovascular disorders, including type 2 diabetes mellitus and atherosclerosis [15,16] and have also been associated with the presence and severity of coronary artery disease [17], suggesting a potential link between this peptide and cardiometabolic dysregulation and vascular pathology.

However, data regarding the role of salusin-β in GDM remain limited, with only a small number of studies directly evaluating salusin levels in pregnancies complicated by GDM [18]. Whether alterations in circulating salusin-β levels reflect endothelial dysfunction, metabolic stress, or adaptive responses to hyperglycemia during pregnancy has not been fully elucidated. Clarifying this relationship may expand current understanding of the vascular and metabolic alterations underlying GDM and may help identify a novel biomarker relevant to cardiometabolic risk in pregnancy.

Accordingly, we hypothesized that circulating salusin-β levels are altered in women with GDM and may have discriminatory capacity for identifying affected pregnancies. Therefore, the present study aimed to compare serum salusin-β concentrations between women with GDM and healthy pregnant controls and to evaluate its diagnostic performance.

## 2. Materials and Methods

All pregnant subjects who participated in the study provided written informed consent. The study protocol was approved by the Ethics Committee of Kanuni Sultan Suleyman Training and Research Hospital (Approval No: 16; Date: 10 January 2019). The study was conducted in accordance with the Declaration of Helsinki.

### 2.1. Study Design and Participants

This prospective study included 144 pregnant women who presented to the Internal Medicine Outpatient Clinic of Kanuni Sultan Suleyman Training and Research Hospital between January and April 2021. Of these, 70 were diagnosed with GDM, and 74 were healthy pregnant controls.

GDM was diagnosed between 24 and 28 weeks of gestation using a 75 g oral glucose tolerance test (OGTT) according to the American Diabetes Association (ADA) criteria. The diagnostic thresholds were defined as any of the following plasma glucose values being met or exceeded: fasting ≥ 92 mg/dL (5.1 mmol/L), 1 h ≥ 180 mg/dL (10.0 mmol/L), or 2 h ≥ 153 mg/dL (8.5 mmol/L).

Eligibility Criteria: Inclusion criteria for both groups were age ≥ 18 years, gestational age between 24 and 28 weeks, no known diagnosis of diabetes prior to pregnancy, and absence of any chronic systemic disease or regular medication use.

Exclusion criteria included any history of cardiovascular, endocrine, renal, hepatic, inflammatory, or autoimmune disease; thyroid dysfunction; chronic pulmonary disease; malignancy; active infection; use of any prescription or over-the-counter medication; and multiple pregnancy. Only otherwise healthy pregnant women without comorbidities and without regular medication use were included in the study.

### 2.2. Clinical and Anthropometric Assessment

Detailed demographic and clinical data, including age, smoking status, body weight, height, body mass index (BMI), waist and hip circumference, family and personal medical history, and medication use, were recorded. Blood pressure measurements were obtained using standard clinical procedures.

### 2.3. Laboratory Measurements

Venous blood samples were collected after 8–10 h of overnight fasting. Routine biochemical parameters, including fasting plasma glucose, lipid profile, alanine aminotransferase (ALT), aspartate aminotransferase (AST), gamma-glutamyl transferase (GGT), uric acid, and HbA1c levels, were analyzed using an automated biochemical analyzer (Abbott Aeroset 2.0, Abbott Diagnostics, IL, USA).

Serum samples were centrifuged and stored at −80 °C until analysis. All samples were analyzed in the same batch to minimize inter-assay variability.

### 2.4. Serum Salusin-β Measurement

Serum salusin-β levels were measured using a commercially available Human Salusin-β ELISA kit (Cat. No. E1574Hu, BT-Lab, Jiaxing, China) according to the manufacturer’s instructions. All samples were analyzed in duplicate, and the mean value was used for statistical analysis. According to the manufacturer’s specifications, the assay had a detection range of 31.25–2000 pg/mL, a lower limit of detection of 18.75 pg/mL, and acceptable dilution linearity over serial dilutions (1:2–1:16).

### 2.5. Assessment of Insulin Resistance

The Homeostasis Model Assessment of Insulin Resistance (HOMA-IR) was calculated using the following formula:

HOMA-IR = fasting insulin (mIU/mL) × fasting glucose (mmol/L)/22.5.

### 2.6. Sample Size and Power Analysis

No formal a priori sample size calculation was performed because this study was designed as an exploratory biomarker investigation in a population with limited prior data on serum salusin-β levels in gestational diabetes mellitus. Following the reviewer’s suggestion, a post hoc power analysis was performed based on the observed difference in serum salusin-β levels between the GDM and control groups. Using the observed effect size (Cohen’s *d* = 0.77), a total sample size of 144 participants (70 women with GDM and 74 healthy controls), and a two-sided significance level of α = 0.05, the achieved statistical power was 99.6%. In addition, the primary multivariable logistic regression model included four predictors with 70 GDM events, corresponding to an events-per-variable ratio of 17.5, indicating adequate model stability. For ROC analysis, the study included 70 GDM cases and 74 controls, and the observed AUC of 0.754 was significantly different from chance performance. Nevertheless, given the exploratory nature of the study, the findings should be interpreted cautiously and confirmed in larger external validation cohorts.

### 2.7. Statistical Analysis

All statistical analyses were performed using IBM SPSS Statistics version 28.0 (IBM Corp., Armonk, NY, USA) and Python -based statistical tools, Python version 3.12 (Python Software Foundation, Wilmington, DE, USA). Normality of continuous variables was assessed using the Shapiro–Wilk test and visual inspection of histograms.

Continuous variables are presented as median (interquartile range) or mean ± standard deviation, as appropriate. Categorical variables are expressed as numbers and percentages. Between-group comparisons were performed using the Mann–Whitney U test or independent-samples *t*-test for continuous variables and the chi-square or Fisher’s exact test for categorical variables.

To evaluate the independent association between salusin-β and GDM, multivariable logistic regression analysis was performed. The primary model was adjusted for age, BMI, and gravidity. To assess the robustness of the observed association and the potential influence of residual confounding, an expanded sensitivity model additionally included smoking status, family history of diabetes, previous GDM history, ALT, creatinine, LDL cholesterol, HDL cholesterol, and triglyceride levels. Odds ratios (ORs) with 95% confidence intervals (CIs) were calculated. Multicollinearity was assessed using the variance inflation factor (VIF) analysis. The VIF values were 1.049 for salusin-β, 1.067 for BMI, 1.077 for age, and 1.131 for gravidity, indicating no evidence of multicollinearity among the independent variables.

Spearman’s rank correlation analysis was performed to evaluate the relationships between serum salusin-β levels and fasting glucose, HbA1c, HOMA-IR, and OGTT measurements. Correlation coefficients (ρ) and corresponding *p*-values were calculated.

In the primary multivariable model, we adjusted for age, BMI, and gravidity as clinically relevant baseline confounders. Glycemic and insulin resistance-related variables (fasting glucose, HbA1c, HOMA-IR, and OGTT values) were not included in either multivariable model because these parameters are integral components of GDM diagnosis and pathophysiology and may act as mediators rather than true confounders. Including such variables could lead to overadjustment and attenuation of the biological association between salusin-β and GDM.

The diagnostic performance of salusin-β was assessed using receiver operating characteristic (ROC) curve analysis. The area under the curve (AUC) with 95% confidence intervals was calculated, and the optimal cut-off value was determined using the Youden index. Internal validation of the ROC analysis was performed using 1000 bootstrap resamples, and the mean bootstrap AUC with its 95% confidence interval was calculated.

A *p*-value < 0.05 was considered statistically significant.

## 3. Results

A total of 144 pregnant women were included (70 GDM, 74 controls). There was no significant difference between the groups in terms of age (*p* = 0.515) or BMI (*p* = 0.093). Gravidity was significantly higher in the GDM group (*p* = 0.010). Fasting glucose levels were significantly elevated in women with GDM compared to controls (*p* < 0.001). Similarly, HbA1c and HOMA-IR values were significantly higher in the GDM group (*p* = 0.023 and *p* = 0.005, respectively). All OGTT measurements (0 h, 1 h, and 2 h) were significantly higher in the GDM group (all *p* < 0.001). No significant differences were observed between the groups regarding previous GDM history, smoking status, or family history of diabetes (all *p* > 0.05) (Table 1).

Comparison of laboratory findings between the groups is presented in Table 2. Most lipid profile components, including LDL, HDL, triglycerides, and total cholesterol, did not differ significantly between women with GDM and controls (all *p* > 0.05).

Among liver function tests, ALT levels were modestly higher in the GDM group (*p* = 0.024), whereas AST and GGT levels were comparable between groups. Creatinine levels were slightly higher in women with GDM (*p* = 0.048). No significant differences were observed in urea, uric acid, hemoglobin, white blood cell count, platelet count, MPV, sodium, or potassium levels (all *p* > 0.05). (Table 2).

**Figure 1:**
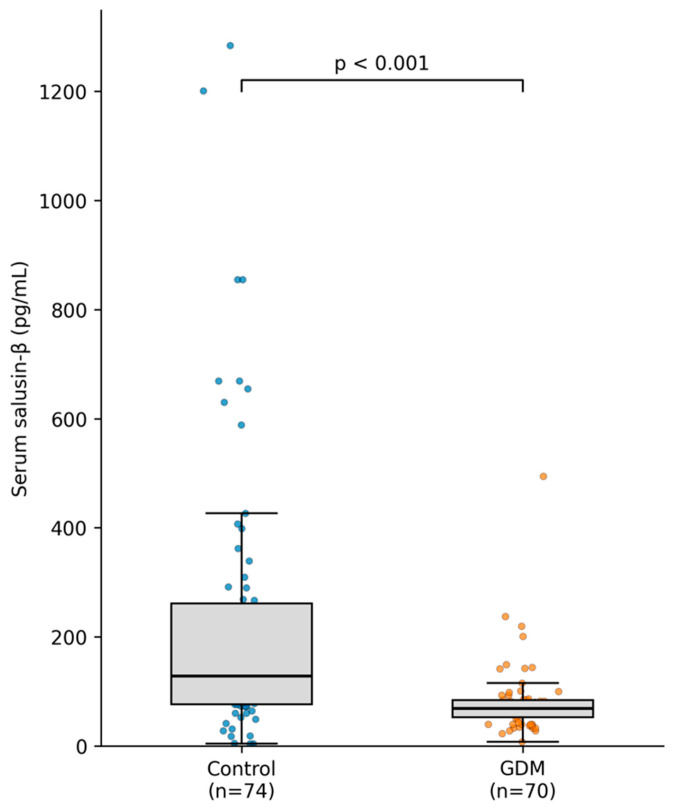
Comparison of salusin-β levels between groups. Boxplots represent the median and interquartile range (IQR), with whiskers extending to 1.5 × IQR. Individual data points are overlaid to illustrate the distribution of serum salusin-β concentrations in each group. Group comparisons were performed using the Mann–Whitney U test.

Salusin-β levels exhibited a right-skewed distribution in both groups (Figure 1). Median salusin-β concentrations were significantly lower in women with GDM compared to healthy controls (68.53 [53.05–84.27] vs. 128.03 [76.74–261.35] pg/mL; *p* < 0.001, Mann–Whitney U test), with a moderate-to-large effect size (r = 0.44) (Figure 1).

In the expanded multivariable logistic regression analysis adjusting for age, BMI, gravidity, smoking status, family history of diabetes, previous GDM history, ALT, creatinine, LDL cholesterol, HDL cholesterol, and triglyceride levels, lower serum salusin-β levels remained independently associated with GDM (OR = 0.990, 95% CI: 0.985–0.996, *p* = 0.001). None of the additional covariates, including demographic, clinical, hepatic, renal, and lipid-related variables, were independently associated with GDM (all *p* > 0.05) (Table 3).

**Figure 2:**
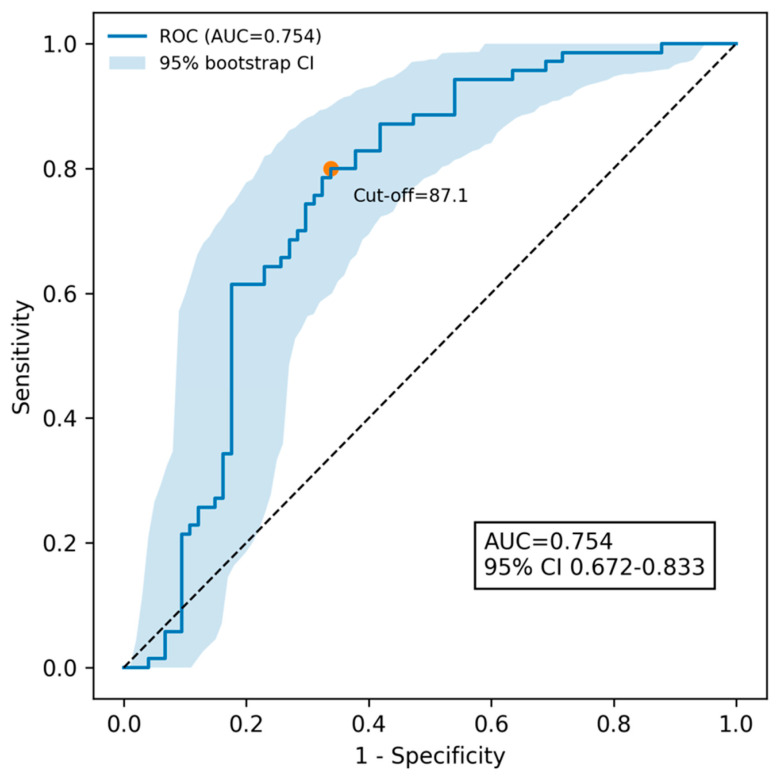
Receiver operating characteristic (ROC) curve of serum salusin-β for identifying gestational diabetes mellitus. Receiver operating characteristic (ROC) curve of serum salusin-β for identifying gestational diabetes mellitus. The shaded area represents the 95% confidence interval obtained by bootstrap internal validation (1000 resamples). The optimal cut-off value determined by the Youden index was 87.1 pg/mL, corresponding to a sensitivity of 80.0% and a specificity of 66.2%. The dashed diagonal line represents the performance of a non-informative classifier.

Receiver operating characteristic (ROC) curve analysis demonstrated that salusin-β had an acceptable ability to discriminate women with GDM from healthy controls (AUC = 0.754, 95% CI: 0.672–0.833). The optimal cut-off value was 87.1 pg/mL, below which the probability of GDM increased. At this threshold, salusin-β showed a sensitivity of 80.0% and a specificity of 66.2%. These findings indicate that reduced salusin-β levels are associated with GDM and exhibit moderate diagnostic performance (Figure 2). Bootstrap internal validation yielded an AUC of 0.754 (95% CI 0.672–0.833).

Spearman correlation analysis demonstrated significant negative correlations between serum salusin-β levels and fasting glucose (ρ = −0.353, *p* < 0.001), HOMA-IR (ρ = −0.272, *p* = 0.001), OGTT 0 h (ρ = −0.413, *p* < 0.001), OGTT 1 h (ρ = −0.416, *p* < 0.001), and OGTT 2 h (ρ = −0.369, *p* < 0.001). No statistically significant correlation was observed between serum salusin-β levels and HbA1c (ρ = −0.147, *p* = 0.079). The correlation coefficients are presented in Table 4, and scatter plots illustrating these relationships are provided in Supplementary Figure S1.

## 4. Discussion

In this prospective study, serum salusin-β levels were significantly lower in pregnant women diagnosed with GDM compared to healthy controls. ROC analysis demonstrated moderate discriminatory performance (AUC = 0.754), suggesting that salusin-β is unlikely to replace OGTT as a standalone diagnostic test but may provide complementary insight into the cardiometabolic alterations observed in GDM. However, based on the observed AUC of 0.754, serum salusin-β should currently be regarded as a complementary research biomarker rather than a clinically applicable standalone diagnostic test. Its relationship with endothelial dysfunction remains hypothetical, as endothelial biomarkers were not evaluated in the present study.

Our findings are broadly consistent with the very limited literature on salusins in GDM; however, important methodological differences should be acknowledged. The study by Celik et al. [18] evaluated both maternal and cord blood salusin levels at the time of delivery in a relatively small cohort that also included SGA pregnancies, whereas the present study specifically assessed maternal serum salusin-β during the standard diagnostic window of GDM (24–28 weeks) in a larger, prospectively characterized population. To our knowledge, evidence on maternal circulating salusin-β in GDM during mid-gestation remains extremely scarce. Importantly, compared with the limited prior literature, our study provides prospective mid-gestational data, ROC-based discrimination analysis, and multivariable adjustment, allowing a more robust evaluation of the independent association between circulating salusin-β and GDM. Therefore, our study extends the limited existing evidence by evaluating the association between serum salusin-β and GDM during the clinically relevant diagnostic window rather than at delivery.

From a mechanistic standpoint, salusin-β has been implicated in vascular inflammation and atherogenic pathways through effects on foam cell formation, vascular smooth muscle cell behavior, and oxidative stress [14]. In parallel, experimental evidence suggests that salusin-β participates in hyperglycemia-related endothelial injury and inflammatory signaling [19,20]. Taken together, the observed reduction in circulating salusin-β in GDM may be consistent with heightened metabolic stress during pregnancy. However, because endothelial function, inflammatory biomarkers, oxidative stress markers, and direct measures of insulin resistance beyond HOMA-IR were not assessed in the present study, these potential biological mechanisms remain hypothetical and cannot be confirmed by our findings. Previous studies have demonstrated endothelial dysfunction in GDM-affected pregnancies [21], and broader evidence indicates that GDM is characterized by immune–metabolic dysregulation rather than isolated hyperglycemia [4,22]. However, our study was not designed to directly evaluate these mechanisms. Accordingly, the involvement of salusin-β within the insulin resistance–inflammation axis remains biologically plausible based on previous experimental evidence, but this hypothesis could not be directly tested in the present study [23].

Importantly, the clinical phenotype of our cohort suggests that the salusin-β difference is not merely a reflection of nonspecific illness severity or concomitant organ dysfunction. The similarity of age and BMI between groups reduces the likelihood of adiposity-driven confounding, and the comparable renal and hepatic biochemical parameters argue against systemic organ impairment as the primary explanation. Rather, these findings suggest a possible association between altered salusin-β levels and GDM-related cardiometabolic alterations. Whether this association reflects endothelial dysfunction or other biological pathways requires further investigation. While gravidity was higher in the GDM group, this is consistent with established obstetric risk profiles for gestational diabetes and represents a potential source of confounding in biomarker studies. Notably, adjustment for gravidity in the multivariable logistic regression did not attenuate the association between salusin-β and GDM, supporting the robustness of the observed biomarker–disease relationship beyond potential obstetric confounding and reducing the likelihood that the findings are solely driven by differences in obstetric history.

Although our AUC suggests moderate discrimination, it does not imply clinical sufficiency as a single-marker test. At present, no universally accepted single biomarker has replaced OGTT in routine GDM diagnosis [24]. A more realistic translational pathway is the incorporation of vascular–metabolic markers into multimarker frameworks to improve risk stratification and diagnostic accuracy. In this regard, evidence that other metabolic peptides such as preptin are altered in GDM supports the concept of complex peptide networks contributing to pregnancy-specific energy and vascular homeostasis [25]. Future work should therefore evaluate whether salusin-β adds incremental value beyond established glycemic indices, ideally using multivariable models, calibration metrics, and external validation. Accordingly, the potential clinical value of salusin-β is more likely to lie in combination with established clinical and biochemical parameters within future multimarker risk prediction models rather than as an isolated diagnostic biomarker.

Future research should focus on validating these findings in larger, multicenter cohorts with diverse ethnic and clinical backgrounds to enhance generalizability. Longitudinal studies incorporating serial salusin-β measurements across different trimesters would be particularly valuable to elucidate temporal dynamics and their relationship with evolving metabolic and inflammatory changes during pregnancy. In addition, future investigations should evaluate the incremental predictive value of salusin-β beyond established glycemic indices using multivariable risk models, calibration analyses, and external validation frameworks. Exploring the combined assessment of salusin-β with endothelial, inflammatory, oxidative stress, and metabolic biomarkers may help clarify the biological significance of salusin-β in GDM. Furthermore, mechanistic studies examining the interaction between salusin-β, insulin resistance, endothelial dysfunction, and placental signaling pathways could provide deeper insight into whether reduced salusin-β levels represent a causal contributor to GDM pathophysiology or a secondary adaptive response to metabolic stress.

In addition, future prospective studies should investigate whether maternal salusin-β concentrations are associated with neonatal outcomes, such as birth weight, neonatal hypoglycemia, macrosomia, neonatal intensive care unit admission, and other adverse perinatal outcomes. If validated, salusin-β may contribute to future multimarker risk assessment strategies alongside routine clinical evaluation rather than replacing OGTT.

## 5. Limitations

Several limitations of this study should be acknowledged. First, although prospectively designed, the study was conducted at a single center in Türkiye, which may limit the generalizability of the findings to populations with different ethnic, genetic, environmental, and clinical characteristics. Since both GDM prevalence and circulating biomarker concentrations may vary across populations, validation in larger multicenter cohorts from diverse geographic regions is warranted. Second, while the sample size was statistically adequate to detect group differences in salusin-β levels, it remains relatively modest for biomarker-based diagnostic modeling and ROC analysis. Although the achieved post hoc statistical power was high for detecting the observed between-group difference in serum salusin-β levels, the possibility of a type II error cannot be completely excluded for secondary analyses, expanded multivariable models, or subgroup analyses because of the relatively modest sample size.

Third, salusin-β concentrations were measured at a single time point during the second trimester; therefore, dynamic longitudinal changes throughout pregnancy could not be evaluated. Given that metabolic, inflammatory, and endothelial responses vary across trimesters, differences in gestational age at sampling may have influenced circulating salusin-β levels.

Fourth, other members of the salusin family, particularly salusin-α, were not assessed, preventing a more comprehensive characterization of the salusin system and peptide interactions in GDM. In addition, potential assay-related variability inherent to ELISA-based measurements should be considered when interpreting biomarker concentrations. Furthermore, endothelial biomarkers, inflammatory mediators (e.g., CRP, IL-6, TNF-α), and oxidative stress markers were not measured. Therefore, any proposed biological mechanisms linking reduced salusin-β levels with GDM should be considered hypothesis-generating rather than directly supported by the present data.

Although internal validation using bootstrap resampling demonstrated stable diagnostic performance, the optimal cut-off value was derived and evaluated within the same cohort. Therefore, external validation in independent populations remains necessary before clinical application.

Finally, given the observational design of the study, causality cannot be established. Whether reduced salusin-β levels contribute directly to the pathogenesis of GDM or represent a secondary epiphenomenon of metabolic and endothelial dysregulation requires further mechanistic and multicenter longitudinal investigations with external validation cohorts.

## 6. Conclusions

In conclusion, serum salusin-β levels were significantly reduced in women with GDM compared to healthy pregnant controls and remained independently associated with GDM after adjustment for demographic, clinical, hepatic, renal, and lipid-related confounders. Although salusin-β demonstrated moderate discriminatory ability, it is unlikely to serve as a standalone diagnostic marker. Instead, it may represent a complementary biomarker with potential value as part of future multimarker risk prediction models, although external validation is required before clinical implementation. Larger, multicenter, and longitudinal studies are needed to further clarify its clinical utility and potential role in cardiometabolic risk assessment during pregnancy.

## Figures and Tables

**Table 1:** Demographic and clinical characteristics of the study population.

Variable	Control (*n* = 74)	GDM (*n* = 70)	*p*-Value
Age (years)	28.00 (24.25–32.00)	29.00 (24.00–33.75)	0.515
BMI (kg/m^2^)	26.59 (23.96–28.52)	26.98 (25.39–31.13)	0.093
Gravidity	2.00 (1.00–3.00)	3.00 (2.00–4.00)	0.010
Fasting glucose (mg/dL)	72.00 (63.00–79.75)	82.50 (76.00–92.50)	<0.001
HbA1c (%)	4.80 (4.53–5.07)	4.95 (4.70–5.30)	0.023
HOMA-IR	1.96 (1.23–2.40)	2.40 (1.71–2.99)	0.005
OGTT 0 h (mg/dL)	82.50 (77.00–85.00)	92.00 (86.25–97.75)	<0.001
OGTT 1 h (mg/dL)	124.00 (108.25–143.75)	180.00 (158.25–195.00)	<0.001
OGTT 2 h (mg/dL)	101.50 (88.00–117.75)	147.50 (131.25–165.75)	<0.001
Previous GDM history, *n* (%)	3 (4.1%)	7 (10.0%)	0.200
Smoking, *n* (%)	10 (13.5%)	10 (14.3%)	1.000
Family history of diabetes, *n* (%)	29 (39.2%)	26 (37.1%)	0.935

Continuous variables are presented as median (interquartile range). Categorical variables are presented as numbers (percentages). Between-group comparisons were performed using the Mann–Whitney U test for continuous variables and the chi-square or Fisher’s exact test for categorical variables, as appropriate. A *p*-value < 0.05 was considered statistically significant.

**Table 2:** Laboratory and biochemical parameters of the study groups.

Variable	Control (*n* = 74)	GDM (*n* = 70)	*p*-Value
LDL (mg/dL)	108.5 (86.8–129.5)	108.5 (81.0–132.8)	0.859
HDL (mg/dL)	68.0 (62.3–79.0)	65.0 (53.3–74.8)	0.195
Triglycerides (mg/dL)	174.5 (139.3–233.3)	198.5 (155.5–246.8)	0.104
Total cholesterol (mg/dL)	215.0 (191.0–244.0)	211.5 (182.5–244.8)	0.848
AST (U/L)	16.0 (14.0–19.0)	17.0 (14.0–20.0)	0.540
ALT (U/L)	12.0 (9.0–14.0)	14.0 (10.0–17.0)	0.024
GGT (U/L)	7.0 (6.0–9.8)	8.0 (5.3–11.0)	0.617
Urea (mg/dL)	13.0 (12.0–17.0)	14.0 (12.0–17.0)	0.211
Creatinine (mg/dL)	0.40 (0.40–0.47)	0.42 (0.40–0.50)	0.048
Uric acid (mg/dL)	3.13 ± 0.63	3.25 ± 0.83	0.315
Hemoglobin (g/dL)	11.30 ± 1.30	11.26 ± 0.95	0.857
White blood cells (×10^3^/µL)	9.5 (8.4–11.1)	10.1 (8.4–11.8)	0.319
Platelet (×10^3^/µL)	235.5 (208.3–289.8)	230.5 (202.3–263.0)	0.273
MPV (fL)	10.80 ± 1.28	10.85 ± 0.94	0.780
Sodium (mmol/L)	137.0 (136.0–138.0)	137.5 (136.0–138.0)	0.909
Potassium (mmol/L)	4.02 ± 0.26	4.14 ± 0.31	0.067

Continuous variables are presented as mean ± standard deviation or median (interquartile range), as appropriate. Categorical variables are presented as numbers (percentages). Group comparisons were performed using the independent-samples *t*-test or Mann–Whitney U test for continuous variables and the χ^2^ test for categorical variables. A *p*-value < 0.05 was considered statistically significant.

**Table 3:** Multivariable logistic regression analysis for the association between salusin-β and GDM.

Variable	OR	95% CI	*p*-Value
Salusin-β	0.990	0.985–0.996	0.001
Age	1.001	0.937–1.071	0.966
BMI	1.066	0.962–1.181	0.224
Gravidity	1.260	0.920–1.726	0.150
Previous GDM history	5.277	0.811–34.356	0.082
Smoking	0.777	0.248–2.434	0.665
Family history of diabetes	0.595	0.262–1.351	0.214
ALT	1.000	0.939–1.066	0.993
Creatinine	90.205	0.429–18,966.702	0.099
LDL cholesterol	1.000	0.988–1.013	0.950
HDL cholesterol	0.985	0.958–1.014	0.306
Triglycerides	0.998	0.992–1.004	0.457

Multivariable logistic regression was performed with GDM status as the dependent variable. Odds ratios (ORs) are reported per one-unit increase in each predictor. The model was adjusted for age, BMI, gravidity, smoking status, family history of diabetes, previous GDM history, ALT, creatinine, LDL cholesterol, HDL cholesterol, and triglyceride levels. A *p*-value < 0.05 was considered statistically significant.

**Table 4:** Spearman correlation analysis between serum salusin-β and glycemic parameters.

Variable	Spearman’s Rho	*p*-Value
Fasting glucose	−0.353	<0.001
HbA1c	−0.147	0.079
HOMA-IR	−0.272	0.001
OGTT 0 h	−0.413	<0.001
OGTT 1 h	−0.416	<0.001
OGTT 2 h	−0.369	<0.001

Spearman’s rank correlation analysis was performed to evaluate the associations between serum salusin-β levels and glycemic parameters. Correlation coefficients (ρ) and corresponding *p*-values are presented. A *p*-value < 0.05 was considered statistically significant.

## Data Availability

The data presented in this study are available from the corresponding author upon request. The data are not publicly available due to ethical and privacy restrictions.

## Supplementary Materials

The following supporting information can be downloaded at: https://www.mdpi.com/article/10.3390/biomedicines14071602/s1, Figure S1: Correlations between serum salusin-β levels and glucose metabolism parameters.
