# Supplementary figures and images for: Decreased Serum Salusin-β Levels Are Independently Associated with Gestational Diabetes Mellitus
Source: Biomedicines. 2026 Jul 17;14(7):1602. doi: 10.3390/biomedicines14071602 (PMC13406535; doi:10.3390/biomedicines14071602)

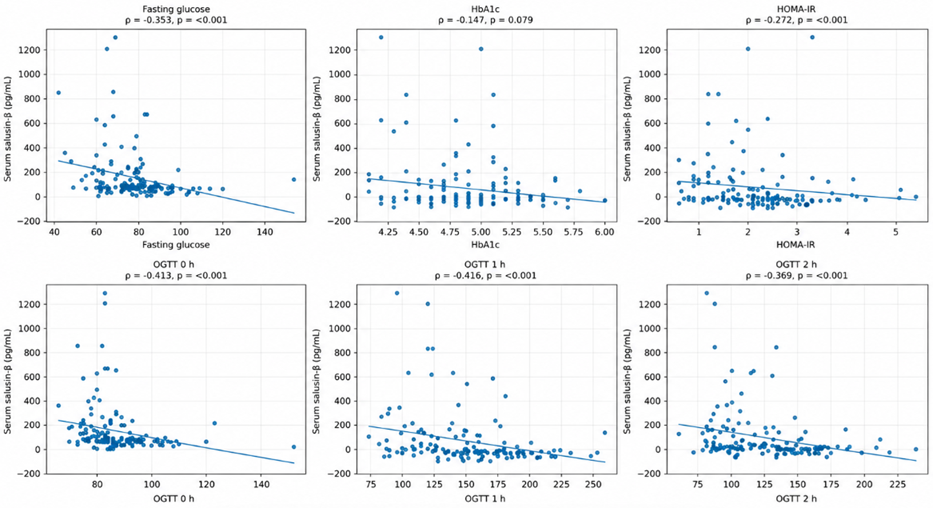

Supplement: Supplementary file 1 [file biomedicines-14-01602-s001.zip › biomedicines-4402562-supplementary.png]
